# Supplementary figures and images for: Comparative chloroplast genomics of three species of Bulbophyllum section Cirrhopetalum (Orchidaceae), with an emphasis on the description of a new species from Eastern Himalaya
Source: PeerJ. 2023 Feb 10;11:e14721. doi: 10.7717/peerj.14721 (PMC9924136; doi:10.7717/peerj.14721)

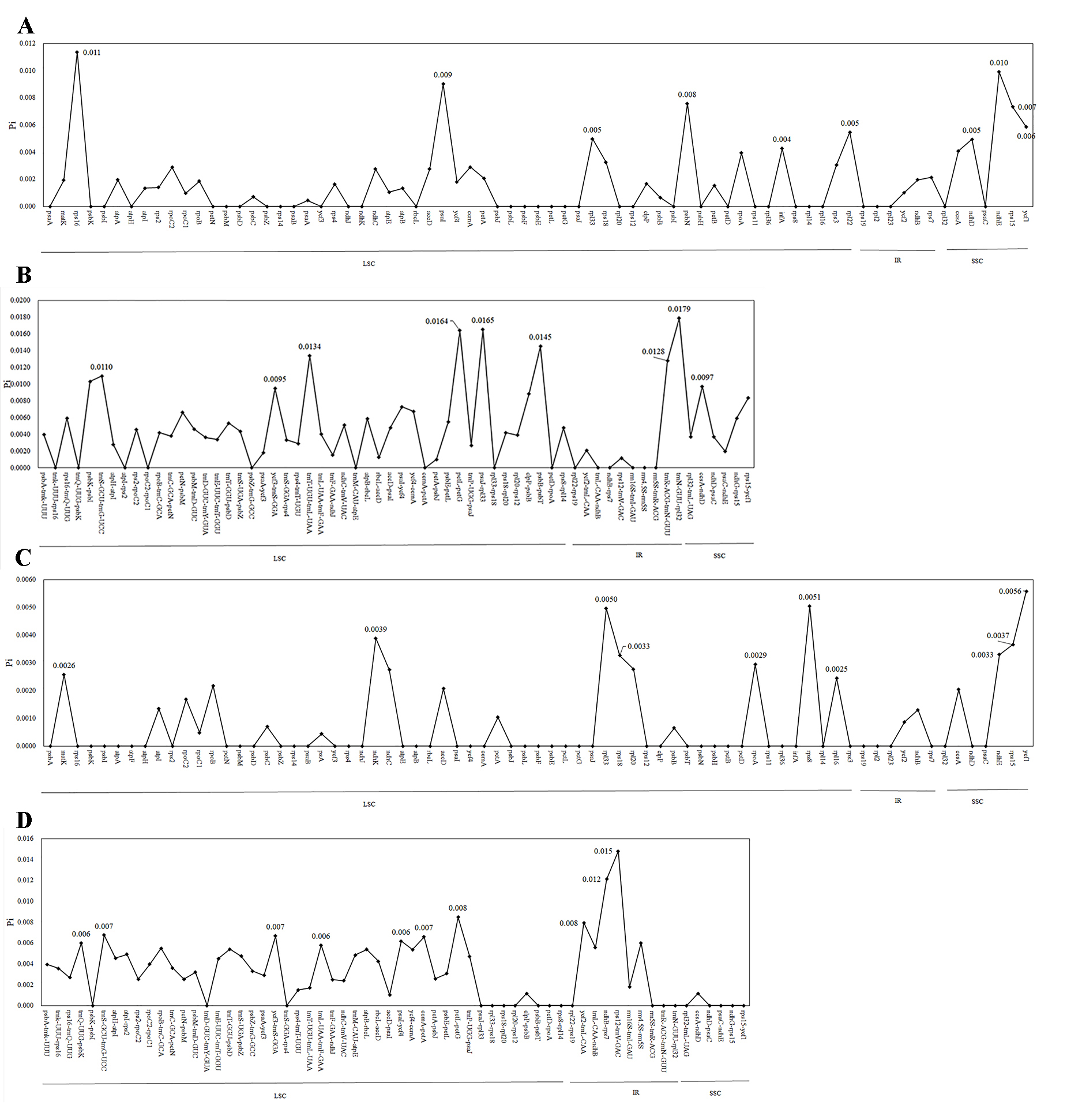

Supplement: Supplemental Information 3 — (A–B) Pi values of coding genes and intergenic regions in the LSC, SSC, and IR regions between B. pilopetalum and B. albociliatum (C–D) Pi values of coding genes and intergenic regions in the LSC, SSC, and IR regions between B. pilopetalum and B. pingnanense [file peerj-11-14721-s003.jpg]
